# Effects of a Family-Based Childhood Obesity Treatment Program on Parental Weight Status

**DOI:** 10.1371/journal.pone.0161921

**Published:** 2016-08-25

**Authors:** Cæcilie Trier, Maria Dahl, Theresa Stjernholm, Tenna R. H. Nielsen, Christine Bøjsøe, Cilius E. Fonvig, Oluf Pedersen, Torben Hansen, Jens-Christian Holm

**Affiliations:** 1 The Children’s Obesity Clinic, Department of Pediatrics, Copenhagen University Hospital Holbæk, Holbæk, Denmark; 2 The Novo Nordisk Foundation Center for Basic Metabolic Research, Section of Metabolic Genetics, The Faculty of Health and Medical Sciences, University of Copenhagen, Copenhagen, Denmark; 3 The Faculty of Health Sciences, University of Southern Denmark, Odense, Denmark; 4 The Faculty of Health and Medical Sciences, University of Copenhagen, Copenhagen, Denmark; Weill Cornell Medical College in Qatar, QATAR

## Abstract

**Objective:**

The aim of this study was to investigate the prevalence of overweight/obesity among parents of children entering childhood obesity treatment and to evaluate changes in the parents’ weight statuses during their child’s treatment.

**Methods:**

The study included parents of 1,125 children and adolescents aged 3–22 years, who were enrolled in a multidisciplinary childhood obesity treatment program. At baseline, weight and height of the parents were obtained by self-reported information and parental body mass index (BMI) was calculated. Weight and height of the children were measured in the clinic and BMI standard deviation scores were calculated. Furthermore, anthropometric data from parents of 664 children were obtained by telephone interview after a mean of 2.5 years of treatment (ranging 16 days to 7 years), and changes in parental BMI were analyzed.

**Results:**

Data on changes in BMI were available in 606 mothers and 479 fathers. At baseline, the median BMI of the mothers was 28.1 kg/m^2^ (range: 16.9–66.6), and the median BMI of the fathers was 28.9 kg/m^2^ (range: 17.2–48.1). Seventy percent of the mothers and 80% of the fathers were overweight or obese at the time of their child’s treatment initiation. Both the mothers and fathers lost weight during their child’s treatment with a mean decrease in BMI in the mothers of 0.5 (95% CI: 0.2–0.8, *p* = 0.0006) and in the fathers of 0.4 (95% CI: 0.2–0.6, *p* = 0.0007). Of the overweight/obese parents, 60% of the mothers and 58% of the fathers lost weight during their child’s treatment.

**Conclusion:**

There is a high prevalence of overweight/obesity among parents of children entering childhood obesity treatment. Family-based childhood obesity treatment with a focus on the child has a positive effect on parental BMI with both mothers and fathers losing weight.

**Trial Registration:**

ClinicalTrials.gov NCT00928473

## Introduction

Several studies have confirmed a correlation between parental and child overweight/obesity, where a higher degree of obesity in parents is associated with a higher risk of obesity in their children [1–4]. Some studies have investigated the prevalence rates of overweight/obesity in parents of obese children undergoing treatment reporting rates as high as 80% [5,6].

Tracking of overweight and obesity through generations may be explained by a number of factors. Several studies have shown that genetic factors contribute significantly to the risk of developing obesity [7,8]. The presence of obese relatives is associated with a higher risk of obesity, even if the family members do not live together or share the same patterns of exercise and food intake [8,9]. Furthermore, environmental factors contribute to this relation, as parental eating habits and eating behavior have been shown to associate with the same habits and behaviors in their children [10]. This underlines the complexity of obesity, where multiple genetic and non-genetic factors interact and affect an increasing number of families, and the need for an intervention strategy integrating the whole family is indeed pertinent [1,2,11,12].

Childhood obesity intervention programs are most often family-based, as children are not expected to implement changes alone. Although the focus in the literature has naturally been on the effects on children [4,13], some studies have targeted both obese children and their overweight/obese parents concurrently and appeared to affect both beneficially [4,14]. Only a few studies have elucidated the effects on parents, whose children are treated for obesity; Wrotniak *et al*. reported parental weight loss as an independent predictor of weight loss in their children during a family-based intervention program involving 142 families [14]; a result that was later supported by two smaller studies [12,15]. Boutelle *et al*. took it a step further and based on a study of 80 parent-child dyads suggested that pediatric weight loss can be achieved by a parent-only approach [16]. Recently, a community-based intervention for the prevention of childhood obesity showed beneficial effects on parental body mass index (BMI) in 478 parents [17]. However, larger studies elucidating the potential effect on parents, whose child is the object of an intervention, are lacking.

In The Children’s Obesity Clinic, Copenhagen University Hospital Holbæk, Denmark, we have shown that 63% of the overweight and obese children and adolescents in the clinic reduced their BMI standard deviation score (SDS) after two years of treatment with a retention rate of 75% [18]. Furthermore, the children improved their metabolic status by decreasing the degree of dyslipidemia [19], 24-hour ambulatory blood pressure [20], and visceral fat and steatosis [21]. The Children’s Obesity Clinic treatment protocol focuses on the child, but one or both parents participate in the consultations together with the child, and the child’s individually tailored treatment plan requires the parent to take active part in the implementation [18]. Therefore, it is plausible that the parents, who are required to alter the living habits of the family, also benefit from these life-style changes alongside their children.

In the present study, we evaluated the prevalence of overweight/obesity among parents of children included in a childhood obesity treatment program. Further, we aimed to investigate changes in parental weight status in the overweight/obese parents during or after their child’s treatment in a childhood obesity treatment center. We hypothesized that this treatment program with previously shown inspiring results in childhood obesity, was also effective in reducing the weight of overweight/obese parents, though they were not the primary objects of the intervention. The aim was thus to elucidate a possible beneficial effect of childhood obesity treatment on parental overweight and obesity.

## Materials and Methods

### Study sample

This study included parents of 1,221 children and adolescents included in the treatment program at the Children’s Obesity Clinic between July 2007 and July 2012. Eligibility criteria for the children were an age of 3–22 years and a BMI above the 90^th^ percentile according to age and sex [22]. Children were referred from their general practitioners, school- and community-based doctors, or pediatric departments nationwide, and there were no other criteria prior to inclusion in the treatment program [23]. For the present study, we further applied an inclusion criterion of minimum two visits to the Clinic. The parents were included as parents of the treated children, and no further eligibility criteria applied to them.

Families with no available baseline BMI data on either parent were excluded (n = 58) as well as siblings of previously included children (n = 38), which left a total of 1,125 families eligible for this study.

### Data collection

Upon inclusion in the treatment program, a pediatrician consulted the family and assessed the child. During the consultation, the pediatrician obtained self-reported current height and weight of both parents as well as information on the family with regards to socioeconomic group (1 to 5 based on parental occupation level [24]), parental marital status, parental custody (if divorced), and the child’s place of residence (with both parents, mainly the mother, mainly the father, or with neither) among others. BMI was calculated as weight in kilograms divided by height in meters squared. For the present study, parents were classified as overweight if their BMI was above 25 kg/m^2^ and obese if their BMI exceeded 30 kg/m^2^. Primary assessment of the child included anthropometric measurements while the child wore light indoor clothes with empty pockets and no shoes. Height was measured on a stadiometer to the nearest 0.1 cm, and weight was measured on a Tanita digital scale (WB-110 MA; Tanita Corp., Tokyo, Japan) to the nearest 0.1 kg. BMI SDS was calculated by the LMS method [25] based on Danish reference charts [22]. At every consultation throughout the treatment, anthropometric measurements of the child were repeated, and for the present study, the child’s last available BMI SDS was used to assess the treatment response.

Baseline data on both children and parents were collected between July 2007 and July 2012, and treatment response data were collected from the children until November 2014. During treatment, the number of visits to the Clinic was registered for each child; each visit being a consultation with either a pediatrician, dietician, nurse, or psychologist.

In March 2011 and again in the period March to May 2013, the parents were contacted by telephone and asked to provide the current height and weight of both parents. We attempted contact with all the families, though some of the children were no longer included in the treatment program. Of the 1,125 families, follow-up data were unavailable in 461 due to lack of contact details, no answer after several attempts, or unwillingness to provide the current data, and they were thus not included in the follow-up analyses.

Informed written and oral consents were obtained from parents or from patients aged 18 years or older. The study is part of the Danish Childhood Obesity Biobank, registered at ClinicalTrials.gov (ID-no.: NCT000928473), is ethically approved by the Ethics Committee of Region Zealand, Denmark, (protocol no. SJ-104), and approved by the Danish Data Protection Agency. The study was carried out in accordance with the Helsinki Declaration of 1975 as revised in 2013.

### Intervention

The children were included in treatment at The Children´s Obesity Clinic, a multidisciplinary, best practice, tertiary childhood obesity treatment clinic encompassing a team of pediatricians, dietitians, psychologists, nurses, social workers, research technicians, and secretaries. The Children’s Obesity Clinic is an accredited European Pediatric Center of Obesity Management by the European Association for the Study of Obesity. At the first visit, a pediatrician presented an individually tailored plan to each child and family comprising 10–25 items of lifestyle changes concerning sources of nutrition, sugar and fat intake, level and type of physical activity, psychosocial functions, eating behaviors, and sleep patterns. The treatment plan was focused on the child; however, the parents were advised that the entire household should adhere to the treatment plan for the child to be able to comply. During the first consultation, obesity and its endocrine regulation were explained in layman’s terms, as the current knowledge on obesity is that endocrinological pathways will counter-adapt to weight loss and seek to promote weight regain by altering energy expenditure, autonomic nervous functions, neuroendocrine functions, and energy intake behaviors towards a higher degree of fat depositioning in the fat cells [18,26]. The family is introduced to the possibility of weight regain during treatment, and the importance of changing multiple habits from the beginning of treatment. Subsequent visits to the Clinic involved consultations with dietitians, nurses, and if required a psychologist and/or social worker. The families attended 30–45 minutes consultations once every six weeks on average, and at each visit the treatment plan was evaluated and optimized. At each consultation, the child and one or both parents attended; however, it was not registered who was present at the consultation. For this reason and because some of the parents were divorced, we opted to perform analyses on mothers and fathers separately.

The children were treated until they reached a BMI below the 75^th^ percentile, attained 22 years of age, voluntarily disengaged from treatment, or dropped out of treatment. The treatment protocol and treatment outcomes have previously been thoroughly described [18].

### Validity of self-reported anthropometric data

In March through November 2011, we included parents in a substudy, where 103 mothers and 52 fathers first self-reported height and weight as usual during the primary consultation and subsequently were measured with the same instruments as the children.

Analyses of these data showed that the mothers overestimated their height by a mean of 1.0 cm (95% CI: 0.7;1.4, *p*<0.0001) and underestimated their weight by a mean of 2.4 kg (95% CI: 1.6;3.1, *p*<0.0001) corresponding to a BMI of 1.2 kg/m^2^ (95% CI: 0.9;1.5, *p*<0.0001) lower than the actual measured value. Similarly, fathers overestimated their height by a mean of 1.7 cm (95% CI: 1.2;2.2, *p*<0.0001) and underestimated their weight by a mean of 2.0 kg (95% CI: 1.0;2.9, *p*<0.0001) corresponding to a BMI difference of 1.1 kg/m^2^ (95% CI: 0.8;1.5, *p*<0.0001).

The correspondences between self-reported and measured BMI in mothers and fathers were evaluated by Bland Altman plots (S1 and S2 Figs) in which there appeared to be no increasing difference in measuring methods with increasing BMI.

### Statistical analysis

Changes in BMI within groups of included parents (all, normal weight, overweight, and obese) were analyzed by paired t-tests, as were the differences between self-reported and measured height, weight, and BMI of the parents in the subanalysis of data validity. The Chi-square test of goodness-of-fit was used to assess equality of the numbers of overweight/obese parents with and without a weight loss. The Chi-square test of independence was used to assess if the fractions of the groups of parents whose children had or had not stopped treatment at the time of parental follow-up were comparable. Differences in parameters between parents included and not included at follow-up, as well as analyses of differences between groups of overweight/obese parents were analyzed with multiple regression analyses adjusted for socioeconomic group, sex of the child, and residence of the child, as these parameters were expected to bias the results. All the multiple regression analyses were further adjusted for number of visits, but as this made no difference in the results, the analyses presented are without this adjustment.

All parameters were analyzed for normality, and where appropriate also for residual normality. If a normal distribution was not found, the parameter was log-transformed prior to further testing. Several parameters involving BMI had a few extreme values (outside double the inter-quartile range), and when testing these, we performed the tests with and without outliers, and as the conclusions remained similar, we presented the results with the outliers.

Statistical significance was set at *p*<0.05. SAS Statistics version 9.4 (SAS Institute Inc., Cary, USA) was used for all statistical analyses.

## Results

Data on changes in BMI were available in 606 mothers and 479 fathers. In 664 children, changes in parental BMI during treatment of the child were available in either the mother or the father, and in 421 of those, the data were available in both parents.

At baseline, the median BMI of the mothers was 28.1 kg/m^2^ (range 16.9–66.6) and the median BMI of the fathers was 28.9 kg/m^2^ (range 17.2–48.1) (Table 1). The median treatment time of their child was 2.5 years for both the included mothers (range: 16 days to 7.0 years) and the included fathers (range: 17 days to 7.0 years) (Table 1). Of the parents included in follow-up analyses, 293 (48%) mothers and 219 (46%) fathers had a child, who was no longer in treatment at the time of the follow-up parental data collection (Table 1). Seventy percent of the mothers and 80% of the fathers were overweight or obese at the time of the treatment initiation (Table 2).

**Table 1:** Descriptive data of the parents of 664 children included in the analyses.

	Mothers	Fathers
**N**	606	479
**Parent baseline BMI, kg/m**^**2**^	28.1 (16.9–66.6)	28.9 (17.2–48.1)
**Child age, years**	11.3 (3.1–22.0)	10.9 (3.1–18.8)
**Child baseline BMI SDS**	2.88 (1.40–5.33)	2.88 (1.40–5.59)
**Child delta BMI SDS**	-0.22 (-3.26–1.32)	-0.27 (-2.66–1.16)
**Child treatment time, years**	2.5 (0.0* - 7.0)	2.5 (0.1** - 7.0)
**Child number of visits**	15 (3–76)	15 (3–76)
**Socioeconomic group** [27]	3 (1–5)	3 (1–5)
**Child sex, percent girls**	57%	59%
**N not in treatment at time of follow-up**	293	219

Data are given as medians with ranges unless stated otherwise.

*16 days

**17 days

**Table 2:** Changes in parental body mass index of the parents of 664 children included in obesity treatment.

	Mothers				Fathers			
	N	% of total	Delta-BMI, kg/m^2^	*p* ^a^	N	% of total	Delta-BMI, kg/m^2^	*p* ^a^
**All**	606	100	-0.5 (-0.8;-0.2)	0.0006	479	100	-0.4 (-0.6;-0.2)	0.0007
**Normal weight**	183	30	0.8 (0.5;1.1)	<0.0001	96	20	0.6 (0.3;0.8)	0.0001
**Overweight**	195	32	-0.4 (-0.7;-0.0)	0.04	191	40	-0.4 (-0.6;-0.1)	0.01
**Obese**	228	38	-1.7 (-2.3;-1.0)	<0.0001	192	40	-0.9 (-1.4;-0.5)	0.0001

Data are given as means with 95% confidence intervals unless stated otherwise.

Normal weight: BMI below 25 kg/m^2^. Overweight: BMI equal to or above 25 kg/m^2^ but below 30 kg/m^2^. Obese: BMI equal to or above 30 kg/m^2^

^a^ Paired t-test was used to calculate the *p*-values.

Both the mothers and fathers lost weight during their child’s treatment with a mean decrease of BMI in the mothers of 0.5 (95% CI: 0.2;0.8, *p* = 0.0006) and in the fathers of 0.4 (95% CI: 0.2;0.6, *p* = 0.0007) (Table 2). When analyzing solely the parents who were obese at baseline, the mean decrease in the BMI of the mothers was 1.7 (95% CI: 1.0;2.3, *p*<0.0001) and of the fathers 0.9 (95% CI: 0.5;1.4, *p*<0.0001) (Table 2). This corresponds to a weight loss of 4.6 kg in a mother of average height (1.67 m), and of 2.9 kg in a father of average height (1.80 m).

Children with at least one overweight/obese parent were more obese at baseline, with a mean BMI SDS of 2.97 (95% CI: 2.91;3.02) compared to 2.75 (95% CI: 2.59;2.90) in children with two normal weight parents (*p* = 0.003). However, there were no differences in the response to treatment when comparing the children of overweight/obese parent(s) (delta BMI SDS: -0.34 (95% CI: -0.39;-0.30)) with those of two normal weight parents (delta BMI SDS: -0.36 (95% CI: -0.54;-0.18)) (*p* = 0.75). These results were independent of socio-economic group, and the children’s age and treatment duration.

Of the overweight/obese parents, 60% of mothers and 58% of fathers lost weight during their child’s treatment (Table 3). There were no differences between the parents with a weight loss and those without (i.e. with stable or increasing BMI) in regards to their child’s age, baseline BMI SDS, treatment response, and treatment duration. Furthermore, the results were independent of socioeconomic group, sex of the child, and residence of the child (Table 3). The overweight/obese mothers who obtained a weight loss were more obese at baseline when compared to those who did not; this was not significant in the fathers (Table 3).

**Table 3:** Differences within the groups of overweight mothers (n = 422) and fathers (n = 386) reported to have obtained a weight loss or no weight loss at follow-up.

	Overweight/obese mothers			Overweight/obese fathers		
	Weight loss	No weight loss	*p*	Weight loss	No weight loss	*p*
**N (% of total)**	255 (60%)	168 (40%)	<0.0001^a^	223 (58%)	160 (42%)	0.001^a^
**N not in treatment at time of follow-up**	120	77	0.78^b^	109	67	0.16^b^
**Parent baseline BMI, kg/m**^**2**^	32.5 (31.8;33.2)	31.2 (30.3;32.0)	0.0008^c^	31.0 (30.5;31.6)	30.7 (29.9;31.5)	0.11^c^
**Parent delta BMI, kg/m**^**2**^	-3.0 (-3.5;-2.6)	1.9 (1.6;2.2)	<0.0001^c^	-2.2 (-2.4;-1.9)	1.5 (1.3;1.8)	<0.0001^c^
**Child age, years**	11.2 (10.8;11.6)	11.3 (10.8;11.8)	0.66^c^	10.7 (10.3;11.2)	10.8 (10.3;11.3)	0.63^c^
**Child baseline BMI SDS**	2.94 (2.86;3.03)	3.05 (2.94;3.16)	0.37^c^	2.89 (2.80;2.98)	3.04 (2.92;3.15)	0.17^c^
**Child delta BMI SDS**	-0.29 (-0.36;-0.22)	-0.38 (-0.48;-0.28)	0.12^c^	-0.40 (-0.47;-0.32)	-0.30 (-0.40;-0.21)	0.14^c^
**Child treatment time, years**	2.6 (2.4;2.8)	2.7 (2.4;2.9)	0.72^c^	2.5 (2.3;2.7)	2.8 (2.6;3.1)	0.22^c^
**Child number of visits**	18.9 (17.3;20.4)	19.0 (17.2;20.8)	0.67^c^	16.6 (15.2;17.9)	19.9 (17.9;22.0)	0.005^c^

BMI, body mass index; SDS, standard deviation score

Data are given as means with 95% confidence intervals unless stated otherwise.

^a^ *p*-values were calculated using the Chi-square test of goodness of fit

^b^ *p*-values were calculated using the Chi-square test of independence

^c^ *p*-values were calculated with multiple regression analyses adjusting for socioeconomic group, sex, and residence of the child. Parent baseline BMI and child treatment time were log-transformed prior to testing due to skewed distributions.

Seventy-two percent of the children of the included mothers and 74% of the children of included fathers decreased their BMI SDS during treatment, which is in line with previous results from our clinic [18]. There were no differences between the overweight/obese parents of children who responded to treatment and of children who did not with regards to parental baseline BMI (32.1 (95% CI: 31.5;32.7) vs. 31.6 (95% CI: 30.4;32.8), *p* = 0.21, in the mothers; 30.8 (95% CI: 30.3;31.3) vs. 31.1 (95% CI: 30.2;32.0), *p* = 0.66, in the fathers) and change in parental BMI during the child’s treatment (-1.0 (95% CI: -1.4;-0.5) vs. -1.3 (95% CI: -2.2;-0.5), *p* = 0.46, in the mothers and -0.7 (95% CI: -1.0;-0.4) vs. -0.5 (95% CI: -1.1;-0.0), *p* = 0.60, in the fathers). The results were independent of socioeconomic group, sex of the child, and residence of the child.

When comparing the mothers included in the analyses of treatment response (n = 606) to those not included (n = 519), there were no differences in maternal baseline BMI (*p* = 0.16) or their child’s change in BMI SDS during treatment (*p* = 0.72) after adjusting for socioeconomic group, the sex of the child, and residence of the child. In the fathers, similar analyses showed no differences in paternal baseline BMI (*p* = 0.16) or in the child’s change in BMI SDS during treatment (*p* = 0.08), when comparing fathers included in follow-up analyses (n: 479) with those not included (n: 646).

## Discussion

In the present study, we showed that 70% of the mothers and 80% of the fathers of children included in childhood obesity treatment were overweight or obese themselves. This prevalence is in concordance with the few studies that have previously investigated this [5,6] and indicates a strong familial influence on childhood obesity, where obesity tracks through generations influenced by both genetic and environmental factors [7,10].

We analyzed parental weight change, though this was not the target of the treatment program, which focuses on the overweight/obese child. Nevertheless, we showed a reduction in BMI in 60% of the overweight/obese mothers and in 58% of the overweight/obese fathers. This is similar to the results of Sato *et al*., who targeted obese adolescents and reported a weight loss in their parents [15]. In the present study, the mean BMI reductions among the obese mothers and fathers were 1.7 and 0.9 kg/m^2^, respectively, and even though the parents were not the focus of the intervention, this level of treatment effect is in fact comparable to lifestyle interventions targeting adults [28].

There were no differences in the weight loss of the children of overweight/obese parents when compared with the children of normal weight parents, and similarly, we found no evidence of an association between the child’s treatment response and parental weight loss. This is partly in contrast to the study performed by Wrotniak *et al*., where they found parental weight change to be a predictor of the child’s weight change in a family-based obesity treatment program [14]. Sato *et al*. found a similar association when analyzing adolescents enrolled in an obesity treatment program [15]. However, both studies showed a decrease in parental BMI during the child’s treatment, which is supporting the results of the present study. Thus, the beneficial weight change of the parent is a positive side effect indicating that targeting children can benefit their parents.

In The Children’s Obesity Clinic, the entire family is invited to participate in the consultations, where obesity and its endocrine regulations are explained (including the context of potential weight regain during treatment) [26]. The families perceive this explanation of obesity as a chronic disease authentic, and can readily relate it to their health identities and perceptions [18,29]. Further, recent qualitative research of this health-pedagogical approach and its effects has investigated the level of participation of the family in the treatment program, and found this to be very important for the child’s (treatment) outcome [30]. The recommended lifestyle changes regarding the child invite the whole family to an alteration in daily habits, and in time, incorporation of these becomes a new lifestyle for the entire family [30].

The anthropometric data on the parents in the present study was self-reported both at baseline and at follow-up, and as such are subject to possible over- or underreporting [31–33]. In our subanalysis of parents who at baseline were measured following their self-report of height and weight, we showed that both mothers and fathers significantly underestimate their BMI, and therefore, the results of the present study should be cautiously interpreted; the number of overweight/obese parents of obese children may be even higher. The follow-up data are subject to the same limitations, and although our substudy indicated no accentuation of misreporting with an increasing BMI, we cannot tell whether there is a change over time, when the child is included in obesity treatment [34]. In future studies, objectively measured anthropometrics in parents throughout the intervention should be implemented.

In addition to the above-mentioned, a limitation of the present study is a possible bias in the assessment of follow-up data, as we were only able to contact 664 of the 1,125 included families. We found no differences in the baseline anthropometric data of the parents included in analyses of treatment effect and those not, as well as no differences in their children’s treatment outcome; however, it is still possible that the parents we were not able to include exhibited a poorer outcome.

Further, the changes in BMI may partially be explained by the phenomenon ‘regression towards the mean’.

Despite the number not included at follow-up, the relatively large number of families in this study is a strength, compared to previous studies of parental weight change which included cohorts of 86 to 153 parents [4,12,14,15].

The positive outcomes in this study may have health and economic benefits for both the parents themselves and for society, as obesity is closely linked to poorer cardiometabolic health; indeed, in our clinic we have previously demonstrated that family members of obese children harbor not only obesity but other cardiovascular comorbidities as well [35]. However, in the future it would be informative to assess whether the parents also benefit from ameliorations in these complications as their children do when treated [18–20].

## Conclusion

The prevalence of obesity among parents of obese children is as high as 80%, but a family-based childhood obesity treatment program with focus on the child has positive effects on parental BMI with both mothers and fathers losing weight during their child’s obesity treatment.

## Supporting Information

S1 Fig Bland-Altman plot of self-reported and measured BMI in mothers in the substudy of validity. (JPG)

S2 Fig Bland-Altman plot of self-reported and measured BMI in fathers in the substudy of validity. (JPG)

S1 File CONSORT Flow Diagram. (DOC)

S2 File Data Set. (XLSX)

S3 File Data on Substudy. (XLSX)
