# Supplementary figures and images for: Effects of a Family-Based Childhood Obesity Treatment Program on Parental Weight Status
Source: PLoS One. 2016 Aug 25;11(8):e0161921. doi: 10.1371/journal.pone.0161921 (PMC4999172; doi:10.1371/journal.pone.0161921)

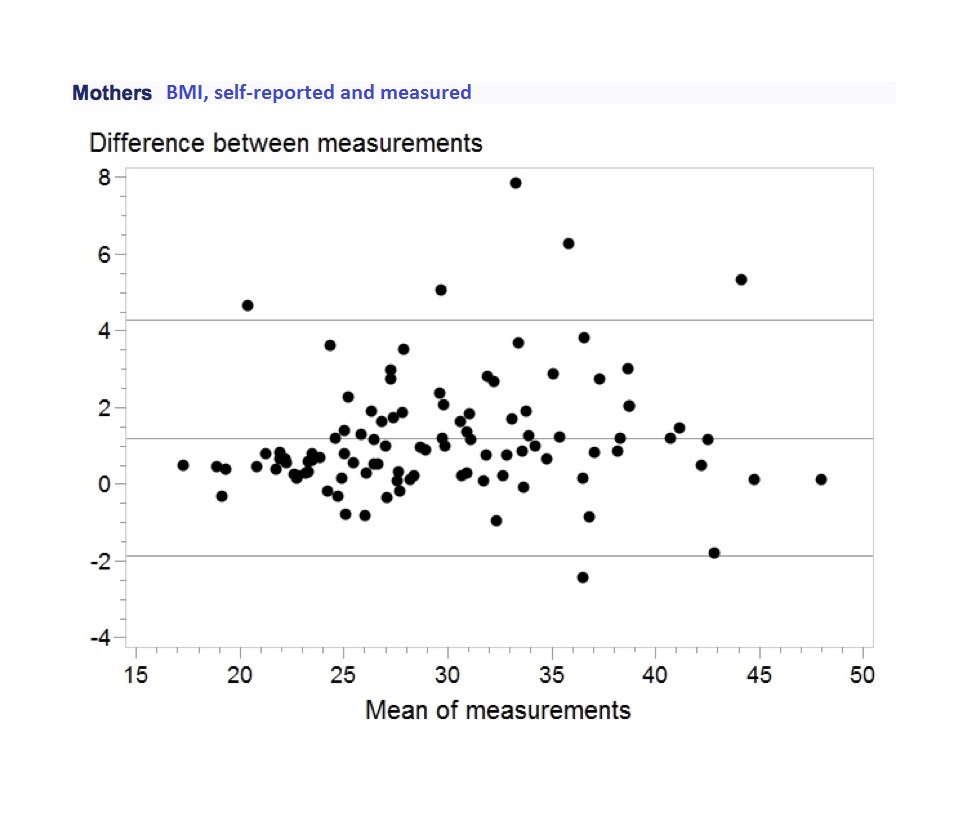

Supplement: S1 Fig — (JPG) [file pone.0161921.s001.jpg]

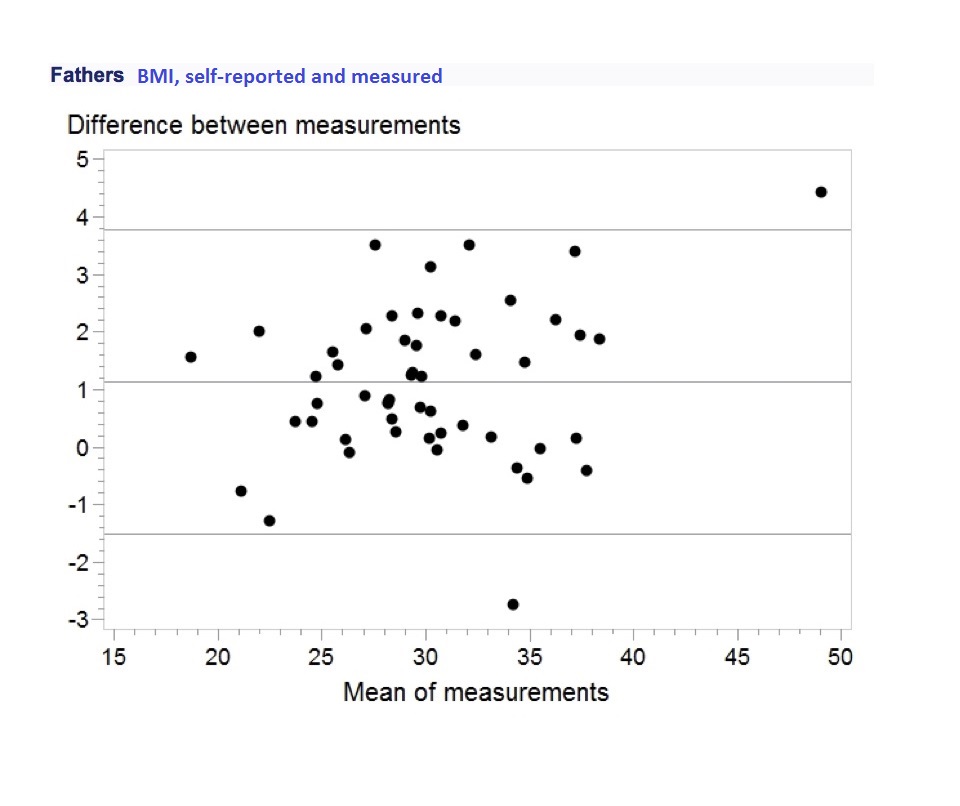

Supplement: S2 Fig — (JPG) [file pone.0161921.s002.jpg]
