# Supplementary material for: Effects of a Family-Based Childhood Obesity Treatment Program on Parental Weight Status
Source: PLoS One. 2016 Aug 25;11(8):e0161921. doi: 10.1371/journal.pone.0161921 (PMC4999172; doi:10.1371/journal.pone.0161921)
Supplement: S1 File — (DOC) [file pone.0161921.s003.doc]

**
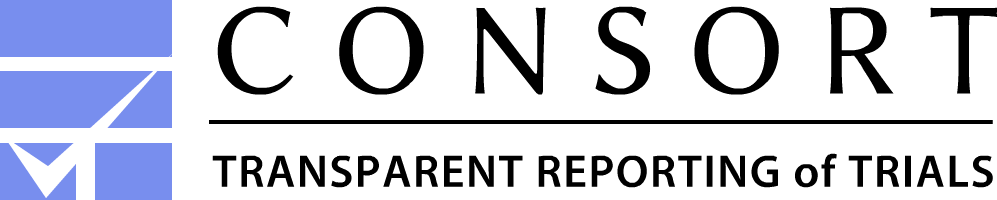
**

**CONSORT 2010 Flow Diagram**

**Allocation**

**Analysis**

**Follow-Up**

**Enrollment**

Assessed for eligibility (n= 1221 families)

Excluded (n= 96)

♦  Not meeting inclusion criteria (n= 58 ), *no baseline BMI on either parent*

♦  Declined to participate (n= 0 )

♦  Other reasons (n= 38), *sibling already included*

Analysed (n= 606 mothers, 479 fathers )
♦ Excluded from analysis (give reasons) (n= 0)

Lost to follow-up (give reasons) (n= 519 mothers, 646 fathers), *no available follow-up BMI*

Discontinued intervention (give reasons) (n= )

Allocated to intervention (n= ), *children received intervention in all included families*

♦ Received allocated intervention (n= )

♦ Did not receive allocated intervention (give reasons) (n= )

Lost to follow-up (give reasons) (n= )

Discontinued intervention (give reasons) (n= )

Allocated to intervention (n= )

♦ Received allocated intervention (n= )

♦ Did not receive allocated intervention (give reasons) (n= )

Analysed (n= )
♦ Excluded from analysis (give reasons) (n= )

Randomized (n= 1125)
